# Development of a one-step multiplex qRT–PCR assay for the detection of African swine fever virus, classical swine fever virus and atypical porcine pestivirus

**DOI:** 10.1186/s12917-022-03144-4

**Published:** 2022-01-18

**Authors:** Huixin Liu, Kaichuang Shi, Jing Zhao, Yanwen Yin, Yating Chen, Hongbin Si, Sujie Qu, Feng Long, Wenjun Lu

**Affiliations:** 1 College of Animal Science and Technology, Guangxi University, Nanning, 530005 China; 2 Guangxi Center for Animal Disease Control and Prevention, Nanning, 530001 China

## Abstract

**Background:**

African swine fever virus (ASFV), classical swine fever virus (CSFV) and atypical porcine pestivirus (APPV) have caused great economic losses to the swine industry in China. Since coinfections of ASFV, CSFV and APPV occur in certain pig herds, it is necessary to accurately and differentially detect these pathogens in field-collected samples. In this study, a one-step multiplex real-time quantitative reverse transcription-polymerase chain reaction (multiplex qRT–PCR) was developed for the simultaneous and differential detection of ASFV, CSFV and APPV.

**Results:**

The one-step multiplex qRT–PCR presented here was able to simultaneously detect ASFV, CSFV and APPV but could not amplify other viruses, including porcine circovirus type 2 (PCV2), pseudorabies virus (PRV), porcine reproductive and respiratory syndrome virus (PRRSV), foot-and-mouth disease virus (FMDV), porcine parvovirus (PPV), porcine epidemic diarrhoea virus (PEDV), transmissible gastroenteritis virus (TGEV), porcine rotavirus (PRoV), porcine deltacoronavirus (PDCoV), border disease virus (BDV), bovine viral diarrhoea virus type 1 (BVDV-1), BVDV-2, etc. The limit of detection (LOD) of the assay was 2.52 × 10^1^ copies/μL for ASFV, CSFV and APPV. A repeatability test using standard recombinant plasmids showed that the intra- and interassay coefficients of variation (CVs) were less than 2%. An assay of 509 clinical samples collected in Guangxi Province, southern China, from October 2018 to December 2020 showed that the positive rates of ASFV, CSFV and APPV were 45.58, 12.57 and 3.54%, respectively, while the coinfection rates of ASFV and CSFV, ASFV and APPV, CSFV and APPV were 4.91, 1.38, 0.98%, respectively. Phylogenetic analysis based on the nucleotide sequences of the partial ASFV p72 gene showed that all ASFV strains from Guangxi Province belonged to genotypes I and II.

**Conclusion:**

A one-step multiplex qRT–PCR with high specificity, sensitivity and repeatability was successfully developed for the simultaneous and differential detection of ASFV, CSFV and APPV.

## Background

African swine fever virus (ASFV) is an enveloped double-stranded DNA virus and the only member of the genus *Asfivirus* in the family *Asfarviridae* [1]. This virus causes African swine fever (ASF), a notifiable disease to the World Organization for Animal Health (OIE) characterized by high fever, extensive haemorrhage, pulmonary oedema and intensive lymphoid tissue necrosis, and it presents high morbidity and mortality [2]. ASF was first identified in Kenya in the 1920s, Europe in 1957, the Caucasus region and southern Russia in 2007 [3, 4], and China in August 2018 [5], where it rapidly spread to most provinces in China within a short time and adversely affected the swine industry [6]. Furthermore, ASF has been reported in other Asian countries, such as Mongolia, Korea, Vietnam, Laos, Cambodia, the Philippines, and Indonesia, since the end of 2018 [7]. ASF was recently reported again in the Dominican Republic in July 2021 (OIE-WAHIS, https://wahis.oie.int/#/report-info?reportId=36844) and Haiti in August 2021 [8], and these reports occurred almost 40 years after the last outbreak of ASF in these countries. ASF has caused severe economic losses to the swine industry worldwide since the 1920s.

Classical swine fever virus (CSFV) is an enveloped single-stranded, positive-sense RNA virus that belongs to the *Pestivirus* genus of the *Flaviviridae* family [9]. CSFV causes classical swine fever (CSF), another notifiable disease of OIE, and it is characterized by high fever, leukopenia, extensive haemorrhage, convulsion and constipation or diarrhoea and presents high morbidity and mortality [10]. CSF was first reported in Ohio, USA, in 1833, and it is still prevalent in many countries worldwide [11, 12]. Although the Chinese C-strain vaccine of CSFV was developed in the 1950s and has been widely used in the field since then, CSF is still sporadic in many regions in China [13, 14]. One explanation for this finding is that many circulating pathogens in China, such as PRRSV and PCV2, can cause immunosuppression [15, 16], which impairs the effect of vaccination with the C-strain vaccine. Another reason is that the circulating CSFVs in the field have high genetic diversity [17, 18], which results in incomplete protection for pig herds even if vaccinated with the C-strain vaccine.

Atypical porcine pestivirus (APPV), a newly discovered virus, is an enveloped single-stranded, positive-sense RNA virus that belongs to the *Pestivirus* genus of the *Flaviviridae* family [19]. It was first discovered in the USA in 2015 [20] and subsequently reported in many other countries in America, Asia and Europe [19, 21]. APPV is a possible causative agent of type A-II congenital tremor (CT) in newborn piglets, which is characterized by generalized body shaking with variable degrees of hypomyelination in the brain and spinal cord [20, 22] and similar to type A-I CT caused by CSFV [23].

ASFV, CSFV and APPV are still prevalent in many countries and cause huge economic losses to the swine industry worldwide. ASF and CSF show similar clinical symptoms and pathological changes, such as high fever, leukopenia, extensive haemorrhage, constipation or diarrhoea, and high mortality [2, 10]. Type A-II CT caused by APPV shows similar clinical manifestations to type A-I CT caused by CSFV in newborn piglets [22, 23]. Therefore, differentiating these diseases in the field is difficult. Furthermore, ASFV, CSFV and APPV were simultaneously prevalent in some countries, and coinfections of these pathogens have been observed in pig herds [24, 25]. Therefore, it is very important to differentially detect these pathogens by laboratory test methods for clinical diagnosis. Currently, several differential polymerase chain reaction (PCR)/reverse transcription (RT)-PCR and real-time quantitative PCR (qPCR)/qRT–PCR assays have been developed for the detection of ASFV [26, 27], CSFV [28, 29], APPV [30], ASFV/CSFV [31, 32] and ASFV/CSFV/APPV [24]. However, a qRT–PCR assay capable of simultaneous and differential detection of ASFV, CSFV and APPV has not been previously reported. Therefore, the objective of this study was to develop a specific, sensitive and reproducible one-step multiplex qRT–PCR for the simultaneous and differential detection of ASFV, CSFV and APPV.

## Results

### Construction of standard recombinant plasmids

The target fragments of the ASFV p72 gene, the CSFV 5′ untranslated region (UTR) and the APPV 5′UTR, were amplified by PCR/RT–PCR, purified and ligated to the pMD18-T vector (TaKaRa, Dalian, China), and then transferred into *E. coli* DH5α competent cells. The positive clones were cultured, and the plasmid constructs were extracted, and then their concentrations were determined by ultraviolet absorbance at 260 nm and 280 nm. The results showed that the original concentrations of the three constructed plasmids, which were named p-ASFV, p-CSFV and p-APPV, were 2.65 × 10^10^ copies/μL, 2.52 × 10^10^ copies/μL, and 3.02 × 10^10^ copies/μL, respectively. These plasmids were used as positive standard plasmids for the optimization of different reaction conditions and for sensitivity and repeatability of the multiplex qRT–PCR.

### Optimal parameters of the multiplex qRT–PCR

After optimization, the reaction conditions, including the annealing temperature, primer and probe concentrations, amplification cycles, etc., were obtained. The reaction mixture of the developed multiplex qRT–PCR was as follows: 10 μL of 2× One Step RT–PCR Buffer III (TaKaRa, Dalian, China), 0.4 μL of Ex Taq HS (5 U/μL) (TaKaRa, Dalian, China), 0.4 μL of PrimeScript RT Enzyme Mix II (RNA/DNA) (TaKaRa, Dalian, China), 0.4 μL of each of ASFV, CSFV and APPV primers (20 pmol/μL), 0.5 μL of ASFV-p72-P (20 pmol/μL), 0.4 μL of CSFV-5′UTR-P (20 pmol/μL), 0.3 μL of APPV-5′UTR-P (20 pmol/μL), 2.0 μL of total DNA/RNA, and distilled water to a total volume of 20 μL. The amplification parameters were as follows: reverse transcription at 42 °C for 5 min; inactivation at 95 °C for 10 s; and 40 cycles of denaturation at 95 °C for 5 s and annealing and extension at 59 °C for 34 s. The fluorescent signals were determined at the end of each cycle.

### Standard curves of the multiplex qRT–PCR

**Fig. 1:**
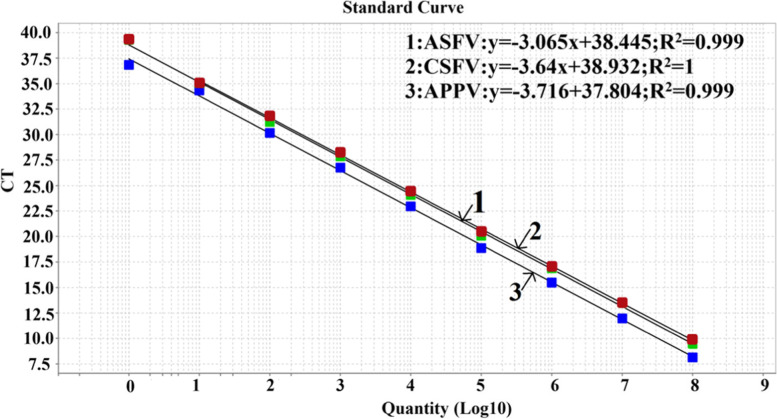
Standard curves of the multiplex qRT-PCR. The triplicate standard curves indicated a linear correlation between the logarithm of the copy number and the Ct values. The concentrations of the standard plasmids (p-ASFV, p-CSFV and p-APPV) ranged from 2.52 × 10^8^ to 2.52 × 10^0^ copies/μL (5.04 × 10^8^ to 5.04 × 10^0^ copies per reaction)

To generate the standard curves of the multiplex qRT–PCR, the standard plasmids of p-ASFV, p-CSFV and p-APPV were mixed together and then serially diluted 10-fold to final concentrations of each plasmid of 2.52 × 10^8^ to 2.52 × 10^0^ copies/μL (5.04 × 10^8^ to 5.04 × 10^0^ copies per reaction). The results showed that the corresponding slope of the equation, correlation coefficient (R^2^), and amplification efficiency (E) were − 3.065, 0.999, and 89.419% for ASFV, respectively; − 3.640, 1.000, and 88.256% for CSFV, respectively; and − 3.716, 0.999 and 85.811% for APPV, respectively (Fig. 1). These results indicated that an excellent linear relationship (R^2^ ≥ 0.999) occurred between the initial template concentrations and the corresponding threshold cycle (Ct) values.

### Specificity of the multiplex qRT–PCR

**Fig. 2:**
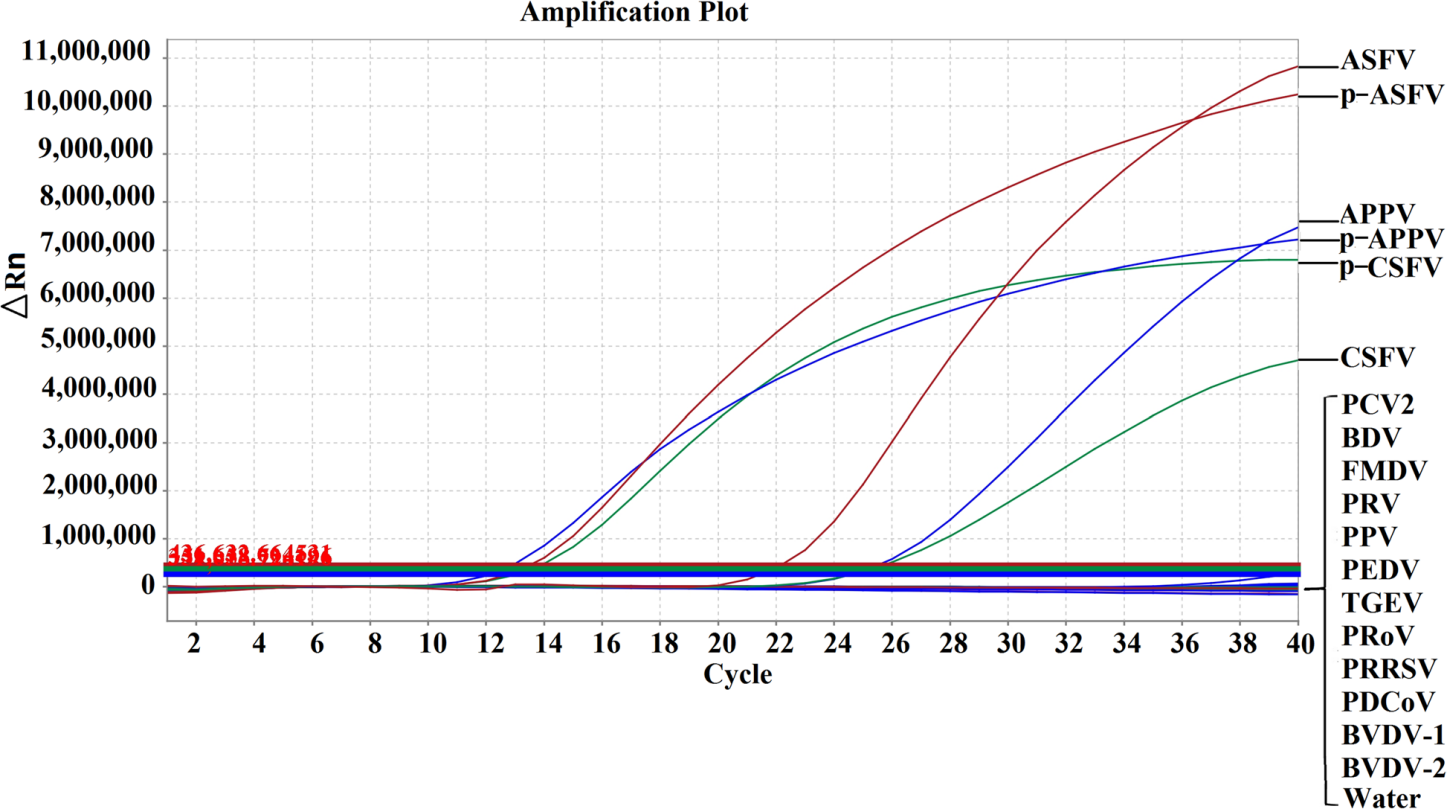
Specificity analysis of the multiplex qRT-PCR using different viral strains. The standard recombinant plasmids (p-ASFV, p-CSFV and p-APPV), ASFV, CSFV, APPV and other viruses (PCV2, PRV, PRRSV, FMDV, PPV, PEDV, TGEV, PRoV, PDCoV, BVDV-1, BVDV-2 and BDV) were used to test the specificity

To evaluate the specificity of the assay, the RNAs/DNAs of ASFV, CSFV, APPV, and 12 other viruses, namely, PCV2, PRV, PRRSV, FMDV, PPV, PEDV, TGEV, PRoV, PDCoV, BVDV-1, BVDV-2 and BDV, were used as templates for multiplex qRT–PCR. The results showed that ASFV, CSFV and APPV had specific amplification curves while the other 12 viruses did not demonstrate any fluorescent signal or amplification curve, indicating high specificity of the assay (Fig. 2).

### Sensitivity of the multiplex qRT–PCR

**Fig. 3:**
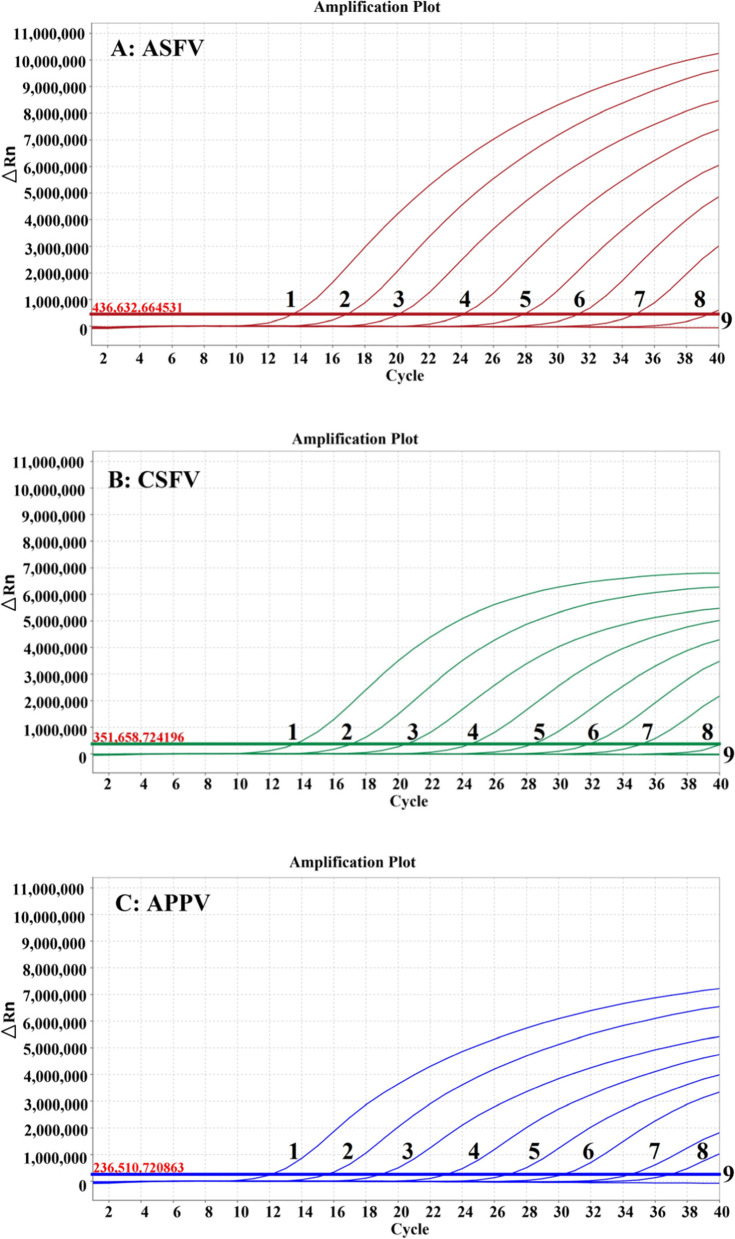
Sensitivity analysis of the multiplex qRT-PCR. The standard recombinant plasmids (p-ASFV, p-CSFV and p-APPV) were used to test the sensitivity. 1-9: 2.52 × 10^7^ - 2.52 × 10^− 1^ copies/μL (final reaction concentrations)

The standard plasmids of p-ASFV, p-CSFV and p-APPV were mixed together and then serially diluted 10-fold from 2.52 × 10^8^ to 2.52 × 10^0^ copies/μL (final reaction concentrations: from 2.52 × 10^7^ copies/μL to 2.52 × 10^− 1^ copies/μL) and used to determine the sensitivity of the multiplex qRT–PCR. The results showed that the limit of detection (LOD) of the assay was 2.52 × 10^0^ copies/μL for ASFV, CSFV and APPV (Fig. 3), while the LOD of the corresponding singleplex qRT–PCR was also 2.52 × 10^0^ copies/μL for ASFV, CSFV and APPV, indicating that the multiplex qRT–PCR had similar sensitivity as the singleplex qRT–PCR. The Ct values of the singleplex and multiplex qRT–PCR are shown in Table 1.

**Table 1:** Comparison of the Ct values between the singleplex and multiplex qRT-PCR

Plasmid	Concentration (copies/μL)	2.52 × 10^7^	2.52 × 10^6^	2.52 × 10^5^	2.52 × 10^4^	2.52 × 10^3^	2.52 × 10^2^	2.52 × 10^1^	2.52 × 10^0^	2.52 × 10^−1^
p-ASFV	Singleplex qRT-PCR	13.781	17.478	20.347	24.044	28.274	31.010	35.073	39.481	–
	Multiplex qRT-PCR	13.846	16.859	20.052	24.052	27.862	31.227	34.776	39.351	–
p-CSFV	Singleplex qRT-PCR	13.490	17.195	20.329	24.584	27.974	31.427	34.793	39.341	–
	Multiplex qRT-PCR	13.788	17.011	20.433	24.418	28.270	31.837	35.074	39.857	–
p-APPV	Singleplex qRT-PCR	12.172	15.430	19.161	23.145	27.183	30.502	33.480	36.519	–
	Multiplex qRT-PCR	11.976	15.469	18.845	22.955	26.767	30.154	34.328	36.830	–

### Repeatability of the multiplex qRT–PCR

To evaluate the repeatability of the assay, three concentrations of 2.52 × 10^7^, 2.52 × 10^5^ and 2.52 × 10^3^ copies/μL (final reaction concentrations) of each standard plasmid in the mixtures were used as templates for the intra- and inter-assay comparisons. The results showed that the intra- and interassay coefficients of variation (CVs) of the Ct values were less than 2% (Table 2), indicating high repeatability of the assay.

**Table 2:** Repeatability analysis of the multiplex qRT-PCR

Plasmid	Concentration (copies/μL)	Ct values of intra-assay			Ct value of inter-asssay		
		$\overline X$	SD	CV (%)	$\overline X$	SD	CV (%)
p-ASFV	2.52 × 10^3^	27.847	0.370	1.329	27.942	0.379	1.356
	2.52 × 10^5^	20.738	0.168	0.810	20.747	0.207	0.998
	2.52 × 10^7^	13.845	0.166	1.199	13.725	0.124	0.903
P-CSFV	2.52 × 10^3^	28.001	0.153	0.546	27.946	0.186	0.666
	2.52 × 10^5^	20.359	0.200	0.982	20.457	0.239	1.168
	2.52 × 10^7^	13.109	0.156	1.190	13.076	0.088	0.673
P-APPV	2.52 × 10^3^	27.328	0.152	0.556	27.314	0.137	0.502
	2.52 × 10^5^	19.125	0.267	1.396	19.128	0.207	1.082
	2.52 × 10^7^	12.348	0.145	1.174	12.302	0.157	1.276

### Detection of clinical samples by multiplex qRT–PCR

A total of 509 clinical samples collected from October 2018 to December 2020 in Guangxi Province, southern China, were detected by the developed multiplex qRT–PCR to evaluate its practicality for the detection of clinical samples. The results showed that the positive rates of ASFV, CSFV and APPV were 45.58% (232/509), 12.57% (64/509) and 3.54% (18/509), respectively, while the coinfection rates of ASFV and CSFV, ASFV and APPV, and CSFV and APPV were 4.91% (25/509), 1.38% (7/509), and 0.98% (5/509), respectively (Table 3). The results detected by the established qRT–PCR were consistent with the results detected by the OIE-recommended real-time PCR/RT–PCR for ASFV and CSFV and the reported real-time RT–PCR for APPV [25] (Table 4). After the test, all samples were treated with high temperature and high pressure as required.

**Table 3:** Detection of clinical samples by the multiplex qRT-PCR

Date	Numbers	ASFV (%)	CSFV (%)	APPV (%)	ASFV+CSFV (%)	ASFV+APPV (%)	CSFV+APPV (%)
Oct, 2018	18	0 (0)	2 (11.11)	1 (5.56)	0 (0)	0 (0)	0 (0)
Nov, 2018	40	0 (0)	9 (22.5)	4 (10.00)	0 (0)	0 (0)	1 (2.50)
Dec, 2018	30	5 (16.67)	6 (20.00)	1 (3.33)	3 (10.00)	0 (0)	1 (3.33)
Jan, 2019	38	5 (13.16)	4 (10.53)	0 (0)	1 (2.63)	0 (0)	0 (0)
Feb, 2019	57	15 (26.32)	5 (8.77)	3 (5.26)	3 (5.26)	2 (3.51)	1 (1.75)
Mar, 2019	36	10 (27.78)	7 (19.44)	0 (0)	4 (11.11)	0 (0)	0 (0)
Apr, 2019	19	12 (63.16)	3 (15.79)	1 (5.26)	0 (0)	0 (0)	0 (0)
May, 2019	16	16 (100.00)	0 (0)	2 (12.50)	0 (0)	1 (6.25)	0 (0)
Jun, 2019	11	4 (36.36)	1 (9.09)	1 (9.09)	0 (0)	1 (9.09)	0 (0)
Jul, 2019	12	12 (100.00)	2 (16.67)	0 (0)	0 (0)	0 (0)	0 (0)
Aug, 2019	28	20 (71.43)	2 (7.14)	0 (0)	2 (7.14)	0 (0)	0 (0)
Sep, 2019	15	15 (100.00)	2 (13.33)	0 (0)	2 (13.33)	0 (0)	0 (0)
Oct, 2019	20	20 (100.00)	1 (5.00)	0 (0)	1 (5.00)	0 (0)	0 (0)
Nov, 2019	24	21 (87.50)	5 (20.83)	3 (12.50)	5 (20.83)	3 (12.50)	2 (8.33)
Dec, 2019	10	6 (60.00)	1 (10.00)	0 (0)	0 (0)	0 (0)	0 (0)
Jan, 2020	10	10 (100.00)	0 (0)	0 (0)	0 (0)	0 (0)	0 (0)
Feb, 2020	10	7 (70.00)	1 (10.00)	0 (0)	0 (0)	0 (0)	0 (0)
Jul, 2020	4	0 (0)	4 (100.00)	0 (0)	0 (0)	0 (0)	0 (0)
Aug, 2020	10	8 (80.00)	0 (0)	0 (0)	0 (0)	0 (0)	0 (0)
Sep, 2020	26	14 (53.85)	2 (7.69)	0 (0)	0 (0)	0 (0)	0 (0)
Oct, 2020	20	13 (65.00)	6 (30.00)	0 (0)	4 (20.00)	0 (0)	0 (0)
Nov, 2020	32	15 (46.88)	1 (3.13)	0 (0)	0 (0)	0 (0)	0 (0)
Dec, 2020	23	4 (17.39)	0 (0)	2 (8.70)	0 (0)	0 (0)	0 (0)
**Total**	**509**	**232 (45.58)**	**64 (12.57)**	**18 (3.54)**	**25 (4.91)**	**7 (1.38)**	**5 (0.98)**

**Table 4:** Agreement between the multiplex qRT-PCR and the reference methods

Detection method	Number of positive samples		
	ASFV	CSFV	APPV
Multiplex qRT-PCR	232/509	64/509	18/509
Reference methods	232/509	64/509	18/509
Agreements	100%	100%	100%

Note: the reference methods refer to the real-time PCR/RT-PCR that was recommended for ASFV (Chapter 3.9.1), CSFV (Chapter 3.9.3) identification by the OIE (OIE Terrestrial Manual 2019) and the real-time RT-PCR for detection of APPV reported by Liu et al. with modification [25]

### Phylogenetic analysis based on ASFV p72 gene

**Fig. 4:**
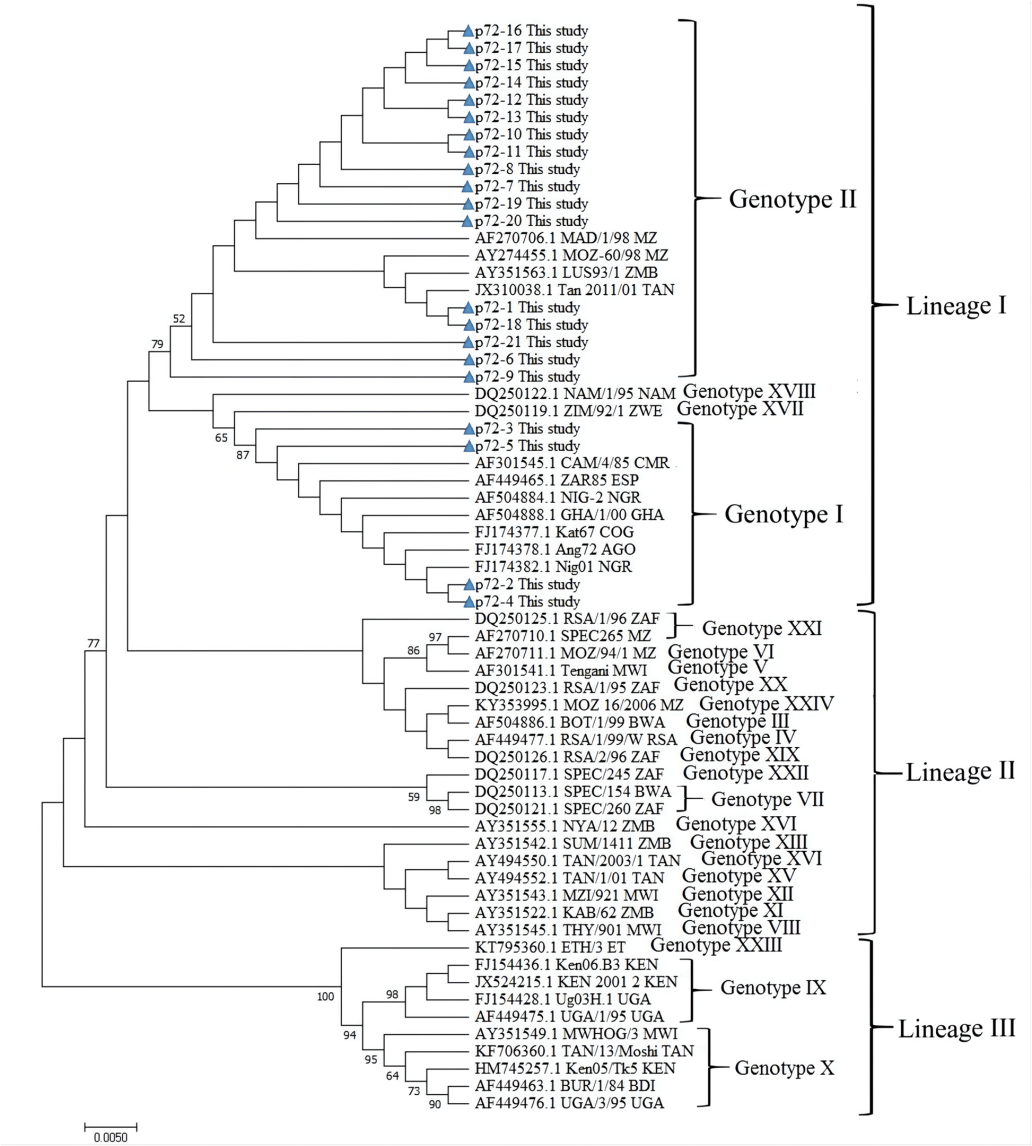
Phylogenetic tree based on partial p72 gene of ASFV. The tree was constructed using the neighbour-joining algorithm of MEGA5.0, and 1000 bootstrap replicates were performed to assign confidences to the groupings. The strains from this study were marked with triangle (△)

A total of 21 clinical samples were selected randomly from the ASFV-positive samples, and the C-terminal end of the B646L gene, which encodes the p72 major capsid protein of ASFV, was amplified and sequenced. Phylogenetic analysis based on partial p72 gene nucleotide sequences showed that 17 strains of the 21 strains obtained from Guangxi Province together with strains from Mozambique (MOZ-60-98, MAD/1/98, MAZ 9/2006), Tanzania (TAN 2011/01) and Zambia (LUS93-1) formed a distinguishable cluster belonging to genotype II, and the other 4 strains from Guangxi Province, together with strains from Nigeria (Nig01, NIG-2), Angola (Ang72), Congo (Kat67), Ghana (GHA/1/00), South Africa (ZAR85) and Cameroon (CAM/4/85) belonged to genotype I (Fig. 4).

## Discussion

ASFV, CSFV and APPV are important pathogens in the swine industry. Coinfection between ASFV and CSFV might occur occasionally in certain pig herds, and the clinical manifestations and pathological changes between these viruses might be hard to distinguish in the field; moreover, similar phenomena are observed between CSFV and APPV [2, 10, 21, 22, 24, 25]. Highly virulent ASFVs of genotype II were first identified in China in August 2018 and have become the main epidemic genotype [33]. However, two strains of genotype I ASFV were recently identified in China, and one strain showed low virulence and efficient transmissibility in pigs and caused mild onset of infection and chronic disease [34], which increased the difficulty of differentially diagnosing ASF and CSF in the field. Therefore, to accurately diagnose these diseases, it is necessary to differentially detect these pathogens in the laboratory and obtain clinical information. Among the many diagnostic methods, qRT–PCR is undoubtedly one of the best choices because it is a rapid, specific, sensitive and accurate method for the detection of viral nucleic acids and can be conveniently used for the quantification and detection of swine viral pathogens [35, 36]. Due to its high throughput, sensitivity, accuracy and ability to detect several pathogens in one reaction within a very short time, multiplex qRT–PCR has been widely used for diagnostic purposes in veterinary laboratories in China. Therefore, a one-step multiplex qRT–PCR was developed to differentially detect ASFV, CSFV and APPV in this study. The assay could specifically detect ASFV, CSFV and APPV with an LOD of 2.52 × 10^1^ copies/μL for each pathogen, and the intra- and interassay CVs were all less than 2%, thus showing high specificity, sensitivity and repeatability. Finally, the developed assay was used to detect 509 clinical samples to further verify its practicality for the detection of samples collected in the field.

The developed qRT–PCR was used to detect 509 clinical samples from Guangxi Province, southern China, for ASFV, CSFV and APPV. The results showed that the positive rates of ASFV, CSFV and APPV were 45.58, 12.57 and 3.54%, respectively, indicating that these viruses were still widely prevalent in pig herds in southern China. Since ASFV and CSFV can cause huge economic damage to the swine industry, great efforts to prevent and control these viruses are required. Furthermore, the coinfection rates of ASFV/CSFV, ASFV/APPV and CSFV/APPV were 4.91, 1.38 and 0.98%, respectively, indicating that coinfections of ASFV, CSFV and APPV were common in certain pig herds. The results were similar to our previous report [25]. Since coinfection of ASFV and CSFV could exacerbate the manifestations and pathological changes [24], with ASFV potentially suppressing the immune response of pig herds vaccinated with the CSFV vaccine [37], the epidemic situation and economic losses will be aggravated. Moreover, it is very important to accurately detect pathogens and rapidly eliminate infected pigs in the early stage. Wild-type and gene-deleted ASFV strains were recently identified in the field from several provinces in China [38, 39]. To ensure that the established qRT–PCR could detect both of these strains, the B646L gene (p72 gene), which is a conserved region for all ASFVs, was selected as a targeting gene to design the specific primers and probes, and the sequences were further blasted in the NCBI database to ensure their conservation to all ASFVs and their specificity to all other viruses. The results in this study showed the high specificity, sensitivity, repeatability and practicality of the developed qRT–PCR. Therefore, the developed multiplex qRT–PCR in this study could provide a useful tool for the rapid differentiation of ASFV, CSFV and APPV in clinical samples from suspected pigs.

ASF was first identified in China in August 2018 and subsequently reported in other Asian countries [8], and it has caused huge economic losses to the swine industry. Since ASF is a newly emergent disease in China and has spread rapidly across this country [7], it is very interesting to study the genomic characteristics of ASFV. The ASFV genome varies by approximately 170 to 193 kb and encodes 150 to 167 kinds of proteins [40]. Based on partial p72 gene sequences, ASFV strains from different countries are currently classified into 24 genotypes and divided into three lineages [41]. The 21 ASFV strains from Guangxi Province evaluated in this study shared a high level of nucleotide homology (97.3% ~ 100%) and amino acid identity (83.4% ~ 95.1%) with strains from different countries in the world (data not shown). Phylogenetic analysis based on partial p72 gene nucleotide sequences revealed that all ASFV strains from Guangxi Province belonged to two genotypes (genotypes I and II), with most of these strains (17 of 21 strains) grouped into genotype II and the other strains (4 of 17 strains) grouped into genotype I. The results showed that genotype I and II ASFV strains were simultaneously prevalent in Guangxi Province, which increased the complexity of circulating strains and made it harder to prevent and control. To our knowledge, this is the first report showing the prevalence of genotype I ASFV in southern China. To date, two of the 24 currently described ASFV genotypes based on the p72 gene sequence, namely, genotypes I and II, have been reported outside Africa [42]. Recently, genotype I and II ASFVs epidemics were reported in several provinces in China, with genotype I showing decreased virulence and caused mild manifestations and pathological changes [43]. Furthermore, genotype II ASFV strains might enable domestic pigs and wild boars to develop chronic infections and become carriers after recovery [44]. According to the surveillance results in this study, genotype II ASFVs were the main circulating strains in Guangxi Province, although genotype I ASFVs were also prevalent in pig herds. The circulation of different genotypes of ASFV in the field will increase the complexity of the disease and the difficulty of its control and prevention. More attention should be given to clinical surveillance to remain abreast of the molecular characteristics and genetic diversity of epidemic ASFV strains in the field.

## Conclusion

In this study, specific primers and probes were designed according to the genomic sequences of ASFV, CSFV and APPV. After optimizing the reaction conditions, including the primer and probe concentrations, annealing temperature, amplification cycles, etc., a one-step multiplex qRT–PCR with high specificity, sensitivity and repeatability was successfully developed for simultaneous and differential detection of ASFV, CSFV and APPV. The ASFV strains from Guangxi Province belonged to genotypes I and II according to the phylogenetic tree, which was based on the nucleotide sequence of the ASFV p72 gene. Thus, the results indicate that at least two genotypes of ASFV are currently prevalent in Guangxi Province, southern China.

## Methods

### Viruses and clinical samples

CSFV (C vaccine strain), PCV2 (SX07 vaccine strain), PRRSV (TJM-F92 vaccine strain), FMDV (O/Mya98/XJ/2010 vaccine strain), PRV (Bartha-K61 vaccine strain), PPV (N vaccine strain), PEDV (CV777 vaccine strain), TGEV (H vaccine strain), and PRoV (NX vaccine strain) were stored in our laboratory. ASFV-, APPV-, BVDV-1-, BVDV-2-, BDV- and PDCoV-positive clinical samples were collected in the field, confirmed by PCR/RT–PCR and gene sequencing, and stored in our laboratory.

A total of 509 clinical samples, including brain, lung, liver, spleen and lymph nodes from each dead pig, were collected from different pig herds in Guangxi Province, southern China, from October 2018 to December 2020. All clinical samples were stored at − 80 °C until used.

### Primers and TaqMan probes

Three pairs of specific primers and corresponding TaqMan probes used for multiplex qRT–PCR assays were designed using Primer Express 3.0 software (ABI, USA) based on the genomic sequences of ASFV (GenBank accession number NC_001659), CSFV (NC_002657) and APPV (KY624591), with a 79 bp fragment amplified for the ASFV p72 gene, a 72 bp fragment amplified for the CSFV 5′UTR and a 90 bp fragment amplified for the APPV 5′UTR. The sequences of the designed primers and probes were analysed using the Blast tool from the National Center for Biotechnology Information (NCBI) and information on published sequences to confirm the high conservation of primers and probes among different reference strains of ASFV, CSFV and APPV. Detailed information on the primers and probes is listed in Table 5.

**Table 5:** Primers and probes used for detection of ASFV, CSFV and APPV

Primer and probe	Sequence (5′ → 3′)	Product size (bp)
ASFV-p72-F	GGCGTATAAAAAGTCCAGGAAATTC	79
ASFV-p72-R	TTCGGCGAGCGCTTTATC	
ASFV-p72-P	Texas Red-TCACCAAATCCTTTTGCGATGCAAGCT-BHQ2	
CSFV-5’UTR-F	CCTGAGTACAGGACAGTCGTCAGT	72
CSFV-5’UTR-R	CCCTCGTCCACATAGCATCTC	
CSFV-5’UTR-P	JOE-TTCGACGTGAGCAGAAGCCCACC-BHQ1	
APPV-5’UTR-F	GGCGTGCCCAAAGAGAAAT	90
APPV-5’UTR-R	GGCACTCTATCAAGCAGTAAGGTCTA	
APPV-5’UTR-P	FAM-TCGGGTCCACCATGCCCCTTT-BHQ1	

### Extraction of nucleic acid

All vaccine viruses and the pooled clinical tissue homogenates (20%, W/V) were resuspended in phosphate-buffered saline (PBS, pH 7.2), vortexed and centrifuged at 12,000×g at 4 °C for 5 min. Total RNA and DNA were extracted from the supernatants using MiniBEST RNA/DNA Extraction Kit Ver. 5.0 (TaKaRa, Dalian, China) according to the manufacturer’s instructions and stored at − 80 °C until use.

### Construction of standard plasmids

Total DNA was extracted from ASFV-positive samples, and total RNA was extracted from CSFV vaccine- and APPV-positive samples and then reverse transcribed to cDNA. The target fragments of ASFV, CSFV and APPV were amplified by PCR using ASFV DNA and CSFV and APPV cDNA as templates. The amplicons were purified and cloned into the pMD18-T vector (TaKaRa, Dalian, China) and transferred into *E. coli* DH5α competent cells (TaKaRa, Dalian, China). The positive clones were cultured at 37 °C for 18 h-20 h and extracted by a MiniBEST Plasmid Extraction Kit Ver. 5.0 (TaKaRa, Dalian, China) for the plasmid constructs. The plasmids were named p-ASFV, p-CSFV and p-APPV and stored at − 80 °C until use as standard plasmids.

The standard plasmids were quantified by ultraviolet absorbance at 260 nm and 280 nm with a NanoDrop spectrophotometer (Thermo Fisher, USA). The exact copy numbers of plasmids were calculated using the following formula:

$$\mathrm{Plasmid}\;\mathrm{copies}/\mu L=\left(6.02\times10^{23}\right)\times\left(\mathrm X\;\mathrm{ng}/\mu L\times10^{-9}\right)/\mathrm{plasmid}\;\mathrm{length}\;\left(\mathrm{bp}\right)\times660$$

### Optimization of the singleplex qRT–PCR assay

The standard plasmids were mixed together and then serially diluted 10-fold from 2.52 × 10^9^ copies/μL to 2.52 × 10^1^ copies/μL (final reaction concentrations: 2.52 × 10^8^ copies/μL to 2.52 × 10^0^ copies/μL) to optimize the reaction conditions of the singleplex qRT–PCR of ASFV, CSFV and APPV. The reaction mixture contained 2× One Step qRT–PCR Buffer III (TaKaRa, Dalian, China) 10 μL, Ex Taq HS (5 U/μL) (TaKaRa, Dalian, China) 0.4 μL, PrimeScript RT Enzyme Mix II (TaKaRa, Dalian, China) 0.4 μL, each primer 0.1-0.6 μL, each probe 0.1-0.6 μL, plasmid template 2.0 μL and distilled water to a total volume of 20 μL. All reactions were amplified by an ABI QuantStudio™ 6 Real-time System (ABI, USA), and the amplification parameters were as follows: 42 °C for 5 min; 95 °C for 10 s; and then 40 cycles of 95 °C for 5 s and 59 °C for 34 s. The fluorescent signals were determined at the end of each cycle.

### Optimization of the multiplex qRT–PCR assay

Based on the optimal reaction conditions of the singleplex qRT–PCR, the reaction conditions of the multiplex qRT–PCR, including annealing temperature, primer concentrations, probe concentrations, amplification cycles, etc., were further determined by orthogonal experiments.

The reaction mixture contained 10 μL of 2× One Step qRT–PCR Buffer III (TaKaRa, Dalian, China), 0.4 μL of Ex Taq HS (5 U/μL) (TaKaRa, Dalian, China), 0.4 μL of PrimeScript RT Enzyme Mix II (TaKaRa, Dalian, China), 0.1-0.6 μL of the primer and probe mixture with different final concentrations, 2.0 μL of the three standard plasmids (mixed in a ratio of 1:1:1) with different final concentrations as templates, and sterilized distilled water to a final volume of 20 μL. The amplification parameters were as follows: 42 °C for 5 min; incubation at 95 °C for 10 s; and then 40 cycles of denaturation at 95 °C for 5 s and annealing and extension at 59 °C for 34 s. Finally, the fluorescent signals were determined at the end of each cycle. After amplification, a Ct value was assigned to each sample. The final concentrations of primers, probes and amplification conditions were optimized to obtain the maximum ΔRn and minimal C_t_ values using standard plasmids of different dilutions as templates.

### Specificity analysis of the multiplex qRT–PCR

The DNA or RNA of ASFV, CSFV, APPV, PCV2, PRV, PRRSV, PPV, FMDV, PEDV, TGEV, PRoV, PDCoV, BVDV-1, BVDV-2 and BDV was used as templates of the developed multiplex qRT–PCR to verify the specificity of the assay.

### Sensitivity analysis of the multiplex qRT–PCR

The standard plasmids of p-ASFV, p-CSFV and p-APPV were mixed together and then serially diluted 10-fold from 2.52 × 10^8^ copies/μL to 2.52 × 10^0^ copies/μL (final reaction concentrations: 2.52 × 10^7^ copies/μL to 2.52 × 10^− 1^ copies/μL) and used as templates for multiplex qRT–PCR to determine the sensitivity of the assay.

### Repeatability analysis of the multiplex qRT–PCR

The standard plasmids of p-ASFV, p-CSFV and p-APPV were mixed together and then serially diluted 10-fold from 2.52 × 10^8^ copies/μL to 2.52 × 10^0^ copies/μL, and concentrations of 2.52 × 10^8^ copies/μL, 2.52 × 10^6^ copies/μL and 2.52 × 10^4^ copies/μL (final reaction concentrations: 2.52 × 10^7^ copies/μL, 2.52 × 10^5^ copies/μL and 2.52 × 10^3^ copies/μL) were used as templates for the developed multiplex qRT–PCR. The intra-assay was performed in triplicate, and the inter-assay was repeated three times, with an interval of 1 week. The intra- and inter-assay CVs were determined to evaluate the repeatability of the assay.

### Detection of clinical samples by multiplex qRT–PCR

A total of 509 clinical samples were collected from pig farms in Guangxi Province, southern China, from October 2018 to December 2020. Total RNA and DNA were extracted from 20% tissue supernatants using a MiniBEST RNA/DNA Extraction Kit Ver. 5.0 (TaKaRa, Dalian, China) and detected by the developed multiplex qRT–PCR for ASFV, CSFV and APPV. The above templates were also detected by real-time PCR/RT–PCR, as recommended for ASFV (Chapter 3.9.1) and CSFV (Chapter 3.9.3) identification by the OIE (OIE Terrestrial Manual 2019), and detected by real-time RT–PCR, as reported for the detection of APPV with modification [25].

### Phylogenetic analysis based on ASFV p72 gene

Twenty-one samples were selected randomly from the positive ASFV samples to amplify a partial p72 gene using a pair of primers (P72-U: 5′-GGCACAAGTTCGGACATGT-3′, P72-D: 5′-GTACTGTAACGCAGCACAG-3′) as previously described [45]. The PCR products were purified, ligated to the pMD-18 T vector (TaKaRa, Dalian, China) and transferred to *E. coli* DH5α competent cells. The positive clones were selected and sequenced (TaKaRa, Dalian, China), and the acquired sequences were edited by the EditSeq program of DNAstar software and aligned with the reference strains retrieved from GenBank using ClustalW. Phylogenetic reconstruction was conducted using the maximum-likelihood algorithm method (T92 + G). Phylogenetic tree reliability was supported using the Kimura distances and a bootstrap method with 1000 replications.

## Data Availability

All data generated or analyzed during this study are included in this article and are available from the corresponding author on reasonable request.

## Abbreviations

APPV: Atypical porcine pestivirus
ASFV: African swine fever virus
BDV: Border disease virus
BVDV: Bovine viral diarrhea virus
CSFV: Classical swine fever virus
CV: Coefficient of variation
FMDV: Foot-and-mouth disease virus
LOD: Limit of detection
multiplex qRT-PCR: Multiplex real-time quantitative RT-PCR
OIE: The World Organization for Animal Health
PCV2: Porcine circovirus type 2
PDCoV: Porcine deltacoronavirus
PEDV: Porcine epidemic diarrhoea virus
PPV: Porcine parvovirus
PRRSV: Porcine reproductive and respiratory syndrome virus
PRV: Pseudorabies virus
TGEV: Transmissible gastroenteritis virus
PRoV: Porcine rotavirus
RT-PCR: Reverse-transcription polymerase chain reaction
